# A Comparative Study of (Cd,Zn)S Buffer Layers for Cu(In,Ga)Se_2_ Solar Panels Fabricated by Chemical Bath and Surface Deposition Methods

**DOI:** 10.3390/ma13071622

**Published:** 2020-04-01

**Authors:** Dowon Bae

**Affiliations:** 1 Department of Chemical Engineering—Materials for Energy Conversion and Storage (MECS), Delft University of Technology, 2629HZ Delft, The Netherlands; d.bae@tudelft.nl or bae.dowon@yahoo.co.kr; 2 LG Innotek R & D Center, 55, Hanyang Daehak-ro, Sanggrok-gu, Ansan, Gyeonggi 426-791, Korea

**Keywords:** chalcogenides, CIGS PV, chemical bath deposition, chemical surface deposition, (Cd,Zn)S, Cu(In,Ga)Se_2_, solar cell

## Abstract

Scale-up to large-area Cu(In,Ga)Se_2_ (CIGS) solar panels is proving to be much more complicated than expected. Particularly, the non-vacuum wet-chemical buffer layer formation step has remained a challenge and has acted as a bottleneck in industrial implementations for mass-production. This technical note deals with the comparative analysis of the impact on different methodologies for the buffer layer formation on CIGS solar panels. Cd(1-x)ZnxS ((Cd,Zn)S) thin films were prepared by chemical bath deposition (CBD), and chemical surface deposition (CSD) for 24-inch (37 cm × 47 cm) patterned CIGS solar panel applications. Buffer layers deposited by the CBD method showed a higher Zn addition level and transmittance than those prepared by the CSD technique due to the predominant cluster-by-cluster growth mechanism, and this induced a difference in the solar cell performance, consequently. The CIGS panels with (Cd,Zn)S buffer layer formed by the CBD method showed a 0.5% point higher conversion efficiency than that of panels with a conventional CdS buffer layer, owing to the increased current density and open-circuit voltage. The samples with the CSD (Cd,Zn)S buffer layer also increased the conversion efficiency with 0.3% point than conventional panels, but mainly due to the increased fill factor.

## 1. Introduction

One of the most appealing advantages of Cu(In,Ga)Se_2_-based (CIGS) solar cells is the potential to grow CIGS thin films on large-area substrates using an in-line vacuum deposition equipment leading to a high-throughput process [1,2]. However, the non-vacuum wet-chemical buffer layer formation step has acted as a bottleneck in industrial implementations for mass-production, since it prevents true in-line processing [3]. While much effort for the recent laboratory cells has been focused on the development of the buffer layer using various vacuum and in-line deposition processes [4,5,6], the buffer layer formation process for the commercially available large-scale CIGS photovoltaic (PV) panels has still remained at a conventional wet-chemical cadmium sulfide deposition stage [1,7]. Notably, chemical bath deposition (CBD) of CdS is very reproducible and yields good step coverage on any chalcopyrite absorber, including CIGS and CdTe [3,8], which are widely used both in PV and photoelectrochemical applications [9]. 

**Figure 1:**
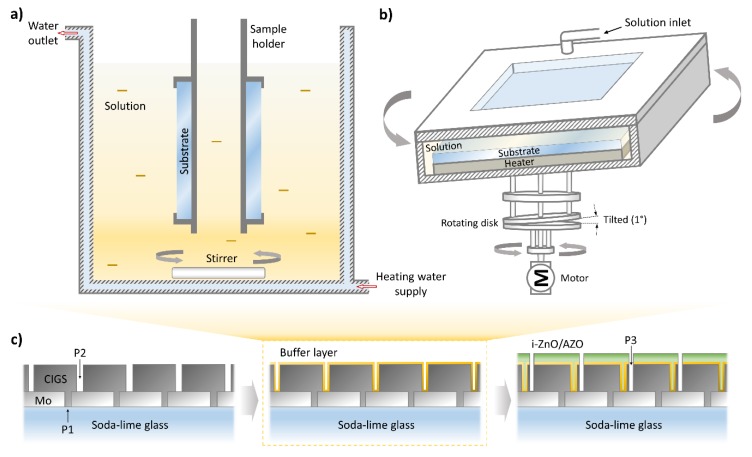
Schematic of apparatus of chemical bath deposition (**a**) and chemical surface deposition (**b**) processes for the buffer layer deposition step during the Cu(In,Ga)Se_2_ (CIGS) photovoltaic (PV) panel fabrication baseline (**c**).

The CBD method additionally takes advantage over other technologies by its large-area deposition capability, which makes the CBD preferable for the PV industry for decades [10]. As shown in Figure 1a, its simple system configuration also makes it the most commonly used method for both industrial and lab-scale solar cells. However, the CBD method requires appropriate back-side protection from unexpected deposition, which can dilute the value of the final products. On the other hand, the chemical surface deposition (CSD) equipment (Figure 1b) induces the chemical reaction via direct heating of the substrate which allows relatively high uniformity of substrate temperature without any back-side contamination. The CSD method is derived from the CBD method, but is differing from the CBD and other methodology by the possibility to obtain a coating of large areas with minimal reagent usage, and, accordingly, minimal number of waste. Owing to these advantages, the CSD CdS process has been introduced for high-efficiency CIGS PV panel manufacturing line, such as Tenuis equipment from Singulus Technologies AG for Manz AG CIGS PV in-line system [11]. In contrast, the easily scalable route of the CSD process is still challenging, while the CBD process shows relatively high scalability due to its batch process, which allows a feasible increase of the panel input per batch. Brief key characters discussed here for both CBD and CSD methods are summarized in Table 1.

Apart from the equipment-wise aspects mentioned above, because of its small bandgap (~2.42 eV), the CdS shows strong absorption in the blue region, i.e., the short wavelength region (≤520 nm), of the solar spectrum, and this results in a parasitic optical loss by this buffer layer [4,12]. Therefore, the alternative n-type materials such as ZnS and Zn-based oxides, which has a broader bandgap with relatively lower toxicity, have been proposed [2]. For instance, a Japan-based company, Solar Frontier, has successfully replaced the CdS buffer layer with a CBD ZnS for its commercial CIGS PV modules [13].

The plain fact is that most solar cell manufacturers choose to set up a mass production line with CBD-based CdS process for the CIGS PV modules because of the relatively poor reproducibility of existing ZnS growth methods (in most cases—chemical solution deposition) [14,15], probably due to low solubility product of ZnS process (i.e., Zn(OH)_2_) resulting in a loose thin-film structure [16,17]. Moreover, the large conduction band offset between CIGS and ZnS also hinders its comprehensive implementation in the industry [12,18]. In this context, Cd_(1-x)_Zn_x_S ((Cd,Zn)S) have stepped into the spotlight of CIGS PV community as a practical “Cd-reduced” buffer layer material because of its wider bandgap, exceeding the bandgap of CdS (~2.42 eV) [19], and its suitability for the CIGS PV has already proven previously [18,19,20]. Although there are numerous research has been reported on the deposition of (Cd,Zn)S buffer layers by wet-chemical deposition [21,22,23], only a few studies discuss the impact of wet-chemical equipment design on the growth behavior of the buffer layer, and CIGS PV panel performance consequently. Notably, no technical report on properties of the (Cd,Zn)S prepared by large area CBD and CSD equipment, which share industrial wet-chemical equipment market for the CISG PV manufacturing system, has been demonstrated. 

The present study is aimed at the comparative investigation on the deposition of a (Cd,Zn)S film on indium tin oxide (ITO) and CIGS PV panel made in pilot production factory using a conventional (i.e., dipping) CBD- and chemical surface deposition (CSD)-type equipment. Optoelectrical and compositional characterizations with statistical analysis for the samples with (Cd,Zn)S prepared using the aforementioned two different types of techniques will be performed. Also, we discuss the operational parameters that merit the most attention in further equipment design towards the mass production line of CIGS PV panels. 

## 2. Materials and Methods

The (Cd,Zn)S thin films were prepared by in-house designed CBD and CSD system, which is schematically illustrated in Figure 1. The CBD equipment in Figure 1a includes a chemical bath with a heating line where a thermostat and a mechanical stirrer provide continuous warm water flow. Mechanically scribed CIGS panels (i.e., after P2 scribing as shown in Figure 1c) were positioned vertically inside the bath using a substrate holder during the reaction. Basically, the CBD system design used in this work is the same as is most commonly used dipping CBD method for fabricating lab-scale CIGS solar cells [20,24]. The cleaned indium–tin–oxide (ITO) coated glass samples were also treated identically with the CIGS panels in order to produce the same buffer layer for optical characterization and thickness monitoring.

On the other hand, the CSD equipment (Figure 1b) includes a direct heating system positioned behind the substrate and a tilted mechanical two-way rotator to ensure uniform thermal and chemical homogeneity throughout the surface. This type of equipment is favorable for providing a thermostated homogeneous substrate with uniform surface temperature. In the conventional dipping CBD process, the heat for driving an activated chemical reaction is transferred from the bath to the sample surface through the solution. In general, the CdS formation reaction is better in the hottest region. Therefore, naturally, the deposition also occurs significantly on the heating mediators, including the bath solution, leading an adhesion of homogeneously produced particles in the bath volume to the film surface. Meanwhile, the CSD process is known to provide a predominant deposition of the buffer layer on the substrate from a heterogeneous growth by an ion-by-ion reaction mechanism [25].

The fabrication sequence of the CIGS PV panel is similar to those demonstrated in our previous reports [7,19,26,27]. We note that the CIGS panels 370 × 470 mm^2^ used in this experiment were fabricated using the first generation pilot line at LG Innotek. A Mo back-contact was deposited onto the soda-lime-glass substrate by sputtering and scribed using a laser ablation process along identically spaced patterns (P1 as shown in Figure 1c). CIGS was sequentially deposited using a three-stage thermal co-evaporation process onto the Mo layer and mechanically scribed (P2). The buffer layer was formed by the above-described wet-chemical methods (CBD or CSD). Finally, the front window layers (i-ZnO/ZnO:Al) were deposited on top of the device with a 40 nm/1μm thickness combination and mechanically scribed (P3). More detailed on device structure and methods (e.g., patterning width, vacuum deposition parameters, etc.) are provided in references [7,19,28]. Indium tin oxide (ITO) coated (100 nm) tempered low-iron glasses (2.8-mm thick) were also used as the substrates for buffer layer thickness and transmittance control. We note that no anti-reflective (AR) layer deposition was applied to the cells in this study.

The confirmed composition of CIGS absorber layer were approximately [Ga]/([Ga] + [In]) = 0.38 ± 0.01 and [Cu]/([Ga] + [In]) = 0.92 ± 0.02, and its thickness was 2.01 ± 0.06 μm. The concentrations of CdSO_4_·xH_2_O, thiourea (NH_2_CSNH_2_), and ZnSO_4_·7H_2_O were 1.1 × 10^−3^, 5.0 × 10^−2^, and 5.1 × 10^−3^ M, respectively. Note that the ZnSO_4_·7H_2_O was not used for the conventional CdS process. Then, the chemicals were dissolved in 2.25 wt. % NH_4_OH mixed deionized water (DI) water. All chemicals used were of reagent grade purchased from Sigma Aldrich. For the CBD process, the starting temperature of the chemical bath was 20 °C, which was then increased up to 80 °C, while the substrate, for the CSD process, was directly heated with the same temperature profile. The thickness of the buffer layer prepared on ITO-glass was determined by using an interferometer (NV-3000, Nanosystem, Daejeon, Republic of Korea). A chemical etching was carried out using a 5 wt. % HCl solution in order to make a step between the ITO and CdS films so that thickness measurements could be easier.

Compositional analysis of the CdS and (Cd,Zn)S layers was performed by scanning electron microscopy combined with energy-dispersive X-ray spectroscopy (SEM-EDX, Nova 200, FEI, Hillsboro, OR, USA). X-ray fluorescence spectroscopy (XRF; Seiko Instruemnts Inc., SFT9500, Inoue, Japan) was used for the thickness and compositional analysis of the CIGS layer. Scanning AES (Auger Electron Spectroscopy, PHI-700, ULVAC-PHI, Chigasaki, Japan) depth profiling was performed on the samples as a supporting tool using a Nanoprobe (PHI 700) system. The formation of CIGS/(Cd,Zn)S/ZnO:Al structure was verified with ex-situ HRTEM (Titan 80-300, FEI, Hillsboro, OR, USA) imaging at an accelerating voltage of 300 kV. Both AES and HRTEM measurements were performed at KIST (Korea Institute Science and Technology) Advanced Analysis Center. The transmittance of the buffer layer was determined by ultraviolet-visible (UV/Vis) spectroscopy measurements (Shimadzu, UV 3600, Kyoto, Japan). The solar cell performance was characterized by current-voltage (J-V) measurements performed by using a solar simulator under AM1.5 equivalent illumination (Wacom Electric, WXS-300S-50, Saitama, Japan). The external quantum efficiency (QE) was also provided to determine the change in the bandgap of the buffer layers used for the working device by measuring the short-circuit current using spectrally resolved monochromatic light (PV Measurements QEX7, Point Roberts, DC, USA).

## 3. Results and Discussion

The thickness, transmittance, and composition of the CdS and (Cd,Zn)S films deposited on ITO-coated glass are shown in Table 2. The deposition time was varied to ensure that the thickness of the (Cd,Zn)S layer was somewhat similar to that of the reference CdS layer (~70 nm) since a large Zn^2+^ concentration can inhibit complex formation between the complexing agent NH_3_ and the Cd^2+^ ion, thus reducing the growth rate of the film [29]. As shown in Table 2, the transmittance of the buffer layer strongly depends on the Zn/(Cd+Zn) ratio of films, whereas the layer thickness remained almost constant. Comparison of CdS and(Cd,Zn)S layers prepared by the CBD reveals that the transmittance of the (Cd,Zn)S layer with Zn/(Cd+Zn) ≈ 0.38 is higher than that of the reference CdS layer by at least 5%~6 % at the same thickness. CdS and (Cd,Zn)S films prepared by CSD were also identified as shown in Table 2. Since the CSD process involves direct heating of the substrate, a relatively short process time was required to ensure a similar level of thickness as those in the CBD process. This reduced process time, along with low chemical usage, would be the principal advantages of making the CSD attractive for the PV manufacturing line. Zn/(Cd+Zn) ratio, as presented in Table 1, obtained by the CSD process shows a slightly lower Zn content than that of similar thickness prepared by the CBD process. The AES depth profile analysis shows reasonably good agreement with the measurement using the EDX used in Table 1 (Figure S1). This compositional discrepancy is probably due to different dominant chemical reaction mechanisms at the surface between the two methods. 

As previously reported [29], the growth of ZnS film is achieved mainly by a cluster-by-cluster mode: ZnS particles form agglomerates, and subsequent accumulation of the ZnS building units results in film formation. In the CBD process, the bath solution is heated up prior to the substrate, and thus, the reaction in the bulk solution is more vigorous than that at the surface, and this may increase the possibility of homogeneous growth by the agglomerated ZnS clusters in the bath. During CSD, on the other hand, the ion-by-ion reaction at the substrate surface is more dominant because of the direct heating from the back-side of the substrate and the flow of the chemical solution along the tilted substrate surface. Overall, the bath condition of the CBD process could be responsible for a favorable environment for the higher Zn-content in the (Cd,Zn)S film grown on the substrate. 

**Figure 2:**
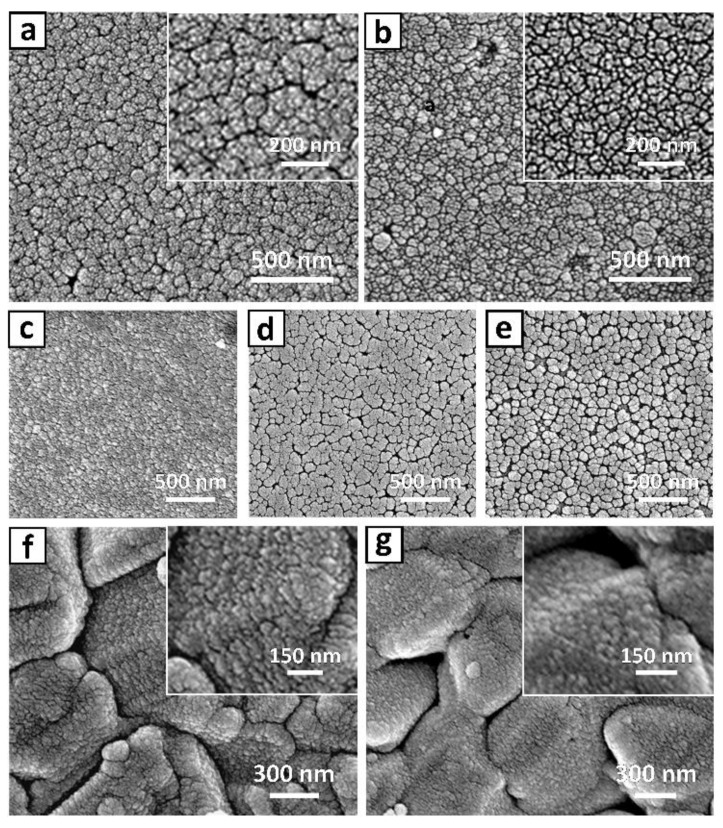
Scanning electron microscopy (SEM) images of samples of as-deposited (Cd,Zn)S films prepared on ITO-coated glass by the CBD (**a**) and CSD (**b**) processes. SEM top-view images of (Cd,Zn)S films obtained from various Zn-concentration (**c–e**) 4.0 × 10^−3^; 1.7 × 10^−2^; and 2.6 × 10^−2^ M, respectively). (**f**) and (**g**) correspond to the (Cd,Zn)S films formed on CIGS surfaces by using CBD and CSD, respectively. Note that the chemical solution for (**f**) and (**g**) is identical to the one used for (**a**) and (**b**).

Figure 2 shows SEM images of the (Cd,Zn)S buffer layer prepared on ITO-coated glass substrates by using CBD and CSD (Figure 2a,b, respectively). As expected, both (Cd,Zn)S films fabricated by CBD and CSD completely cover the glass substrate across the sample area. These images also indicate that the buffer layers have a granular structure with very well defined granular boundaries. Generally, the CdS is known to have a very good match with the ITO glass substrate, as reported previously [8,30]. Nonetheless, the SEM images in Figure 2a indicates the buffer layer prepared by the CBD method has larger granules than the film formed by the CSD method (Figure 2b). Clear and fine boundaries between the granules prepared by the CSD method also support the smaller granular size of the (Cd,Zn)S film. This might be related to the diffusion of Zn into the CdS film, which leads to a larger lattice constant than that of conventional CdS [24]. The lattice constant of wurtzite CdS crystal is about 4.16 Å at 300K, while the ZnS with zinc blende structure has 5.42 Å. Figure 2c–e show the plain-view SEM images of (Cd,Zn)S films with various ZnSO_4_·7H_2_O concentrations (4.0 × 10^−3^; 1.7 × 10^−2^; and 2.6 × 10^−2^ respectively). It was found that the granular size of the (Cd,Zn)S layer increases with increasing the ZnSO_4_·7H_2_O concentration in the chemical solution. An interesting feature is that the number of void in samples also increases with Zn-content in the solution. As expected, dense (Cd,Zn)S buffer layer was also obtained on CIGS thin films (Figure 2f,g). The morphological tendency is quite similar to that on the ITO substrates and the CBD (Cd,Zn)S layer has larger granules with clear boundaries compared to the one prepared using a CSD process. 

However, the EDX analysis (Table 3) revealed that the buffer layer formed on the CIGS photo-absorber by both CBD and CSD under the (Cd,Zn)S conditions showed significantly reduced Zn/(Cd+Zn) ratio (0.04~0.20) in contrast to the films formed on the ITO-coated glass samples (0.28~0.39). In common with other thin film deposition techniques, rough morphologies tend to give more adherent films than corresponding smooth ones. In spite of the fact that glass itself is relatively inert, the surface of the glass can be very reactive towards species in solution. Specifically, the surface of the ITO is hydroxylated in aqueous solution, and the surface hydroxide groups can bind chemically to constituents (e.g., cationic precursors). Moreover, the heavy metal content of the ITO film, as was already shown previously [31], tend to bind with S-containing anionic precursors of the solution.

Interestingly, the EDX analysis in Table 3 reveals the surprisingly low Zn-content in the (Cd,Zn)S layer, which was prepared using a CSD process. The TEM measurement with an energy-dispersive spectroscopy line-scan across the (Cd,Zn)S/CIGS interface (Figure S3) also evidenced that only slight diffusion of Zn into the CdS film could be measured in the case of CSD process. In contrast, the sample prepared by the CBD process showed a noticeable amount of Zn from the buffer layer. This can be explained in the following manner: The hydroxylated ITO in aqueous solution can form fairly strong hydrogen bonds (e.g., =Sn–OH^−^ ↔ =Sn–O^−^ + H^+^), which reduces the local pH that is a favorable condition for the reaction between the [Zn(NH_3_)_4_]^2+^ and S^2−^ in the solution [32,33]. Meanwhile, in the solution with the CIGS sample, the ZnS is formed scarcely due to a relatively slow reaction of the [Zn(NH_3_)_4_]^2+^ with S^2−^. In this case, the CdS nuclei formation takes place on the substrate under the ion-by-ion growth mechanism instead of adhesion of the particles formed by the cluster-by-cluster mechanism in the solution, such as ZnS particles.

**Figure 3:**
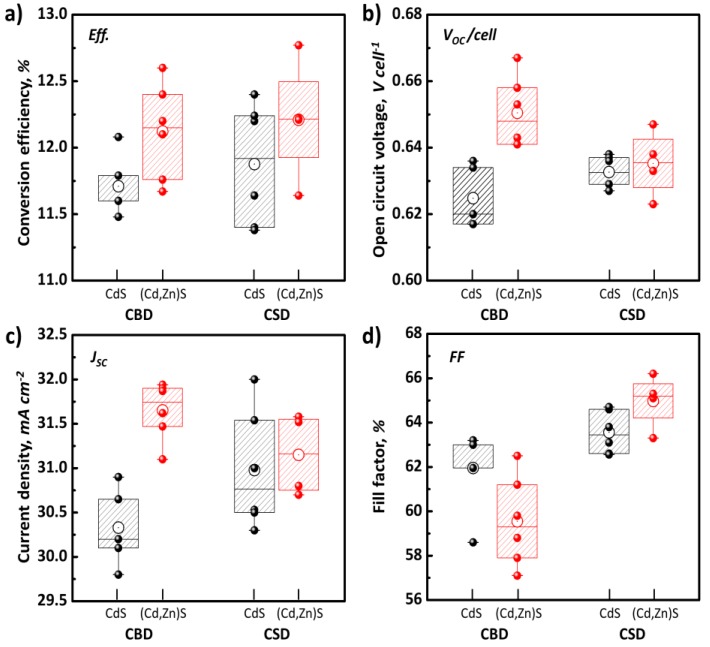
Statistics of photovoltaic current-voltage (J-V) performance parameters of CIGS panels fabricated with various buffer layers (CdS and (Cd,Zn)S prepared by CBD and CSD methods) under simulated illumination of 100 mW/cm^2^ AM 1.5G. (**a**) Conversion efficiency; (**b**) Cell voltage; (**c**) Current density; (**d**) Fill factor.

**Figure 4:**
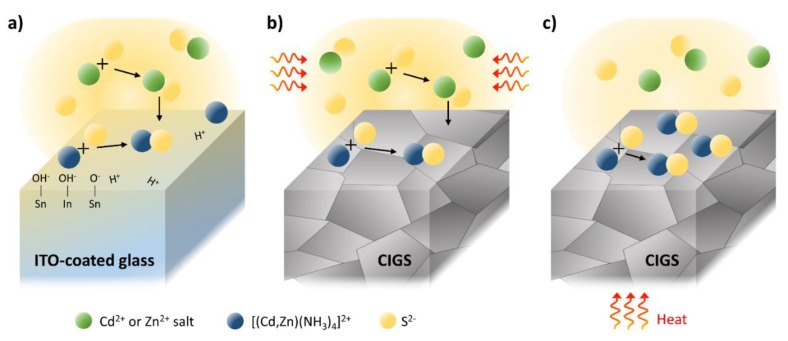
Illustration for the mixed mechanism of the cluster-by-cluster and ion-by-ion growth regimes during the (Cd,Zn)S deposition on the ITO-coated glass (**a**) and on the CIGS substrate during the CBD process (**b**). The ion-by-ion growth dominant mechanism during the CSD process is also illustrated in (**c**).

The *I–V* characteristics of the CIGS PV panels with different buffer layer treatments are shown in Figure 3 (see also Table 4). Irrespective of the deposition method adopted, the cells fabricating using the (Cd,Zn)S solution showed comparatively higher efficiency (0.3–0.4% absolute point) than did those prepared using the CdS buffer layer. Similar to the behavior on the ITO surface (Figure 4a), it is also assumed that the Zn-salts react with S^2+^ ions in the solution under cluster-by-cluster growth regime and are integrated into the CdS which is formed under a mixed growth regime (cluster-by-cluster and ion-by-ion growth mechanism (Figure 4b)). We emphasize that all CIGS panels used in this study were obtained from the same batch so that we can focus on parameters merit the most attention, i.e., effect of the buffer layer on the PV performance. In the case of the (Cd,Zn)S-based cells formed by CBD, the improvement in efficiency is attributed to the increase in both open-circuit voltage (*V_OC/cell_*) and current density (*J_SC_*). This result well coincides with the previous report that Zn addition increases the ban-gap of the buffer layer. It thus results in lower optical loss from the buffer layer (i.e., better blue photon response) and a suitable conduction band offset with CIGS light-absorber layer due to a reduced conduction band discontinuity at the (Cd,Zn)S/CIGS junction [34,35,36]. Hamri et al. [36] revealed in recent theoretical work that the open-circuit voltage (*V_OC_*) slightly increases by the change on the Zn concentration from 0 to 0.6 (relative to the Cd), above which the presence of spike at the interface hinders a feasible electrons transfer from the CIGS. 

**Figure 5:**
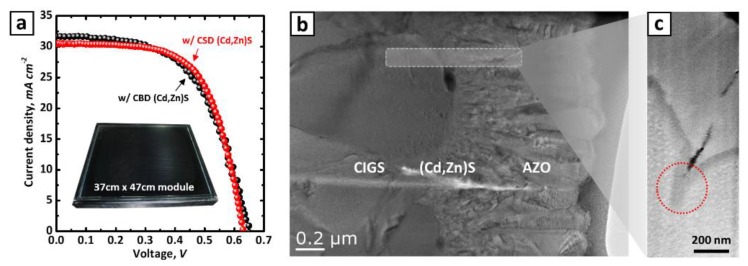
J–V characteristics of CIGS panel with various buffer layers (dark – CBD (Cd,Zn)S; red – CSD (Cd,Zn)S) (**a**). TEM cross-sectional image of the CIGS PV cell with a (Cd,Zn)S buffer layer deposited by a CSD process (**b**) and zoomed-in focused ion beam (FIB) SEM image for the interface at CIGS/(Cd,Zn)S (**c**).

In the case of the CIGS PV panels with the (Cd,Zn)S-based buffer layer formed by CSD, the efficiency gain was approximately 0.34 % absolute point, which is mainly resulted from the increased fill factor (*FF*). It is obvious that the increase in *FF* is primarily attributed to the improved shunt resistance (*R_sh_*), as the series resistance (*R_s_*) remained nearly unchanged (see Table 4). Naturally, *J_sc_* remained nearly unchanged (Table 4), as the EDX results in Table 3 revealed a very low Zn/(Cd+Zn) ratio for the buffer layer implying that any meaningful optical bandgap of the buffer layer cannot be observed. This different trend PV performance for the cell with a CSD (Cd,Zn)S buffer layer towards the one with CBD (Cd,Zn)S layer is well-represented in the *J-V* curves (Figure 5a). As shown in Table 2, the processing time required to form a (Cd,Zn)S layer with the same thickness to that of the reference CdS layer (~70 nm) was 4 min longer than the established time for the reference CdS layer formation. This increased process time may influence the stack-coverage by the buffer layer, probably arising from a prolonged time to sufficiently cover the rough surface with the chemically-grown particles on the CIGS surface under the ion-by-ion growth regime, as illustrated in Figure 4c. 

The SEM image of the (Cd,Zn)S formed on a CIGS layer by using the CSD method (Figure 2g) confirms a conformal and much smoother coverage compared to the one formed by CBD (Figure 2f). This conformal coverage by the buffer layer is also well demonstrated in the TEM and SEM images shown in Figure 5b,c. As indicated with a red circle in Figure 5c, the crevice between the CIGS grains also is well protected by a dense buffer layer. Though the present experimental results do not directly support the hypothesis mentioned above, it is a well-established fact that pinholes or voids resulted from inadequate surface coverage create a shunting path that contributes to the decreased *R_sh_*, which is intrinsically related to the *FF*, as has been argued previously [19,22,37]. 

**Figure 6:**
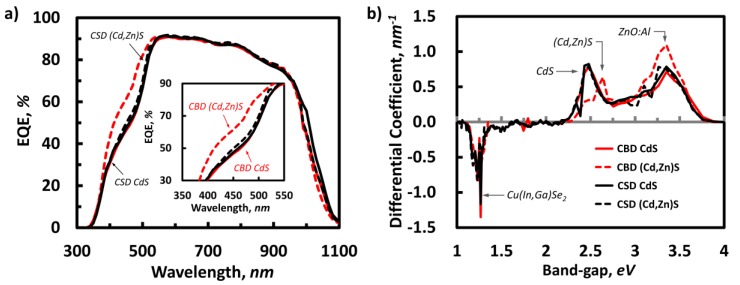
External quantum efficiency (EQE) (**a**) and differential EQE (**b**) graph for the CIGS PV panels with different type of buffer layers (red – CBD and black – CSD).

As evidenced in external quantum efficiency (EQE) measurements (Figure 6a), an improved response from the (Cd,Zn)S buffer layers prepared by a CBD process (from 350 to approximately 520 nm in wavelength) also well agrees with the abovementioned description. The differential EQE (dEQE(λ)/dλ) conversion (Figure 6b) also yields a positive peak shift for the buffer layer (from 2.5 to 2.7 eV) which is attributable to a slight bandgap shift due to high Zn concentration in (Cd,Zn)S layer. Meanwhile, the overlapping characteristic peaks from the differential EQE for other buffer layer cases reveal the steady EQE responses without any noticeable bandgap shift. Another interesting feature specific to the CBD (Cd,Zn)S buffer layer case is the decrease in fill factor (*FF*) as plotted in Figure 3d. In connection with the increased series resistance (*R_s_*) in Table 4, the (Cd,Zn)S buffer layer with a high Zn/(Cd+Zn) ratio might increase the resistivity of the buffer layer, and the lowered *FF* consequently. As reported previously [38,39], the resistivity of the (Cd,Zn)S films increases with increasing Zn-content due to decreasing carrier density of the (Cd,Zn)S layer. 

## 4. Conclusions

(Cd,Zn)S layers were grown by chemical deposition using CBD and CSD processes. The transmittance and growth rate of the (Cd,Zn)S buffer layers on the ITO-coated glass were directly and inversely proportional to the Zn addition level, respectively. CIGS solar panels with (Cd,Zn)S buffer layers deposited by both CBD and CSD processes have shown improvement in conversion efficiency. In particular, the conventional CBD process with Cd and Zn components led to increased cell efficiency because of the increase in *J_SC_* and *V_OC_*. However, the use of CSD with the same solution led to improved cell efficiency because of the increased *FF*, while the *J_SC_* remained unchanged. EDX and QE results confirmed that the concentration of the Zn component incorporated into the CdS layer differs depending on the surface type. Unlike the CBD buffer layer prepared on the CIGS PV panels, the relatively small quantity of the Zn was found from the (Cd,Zn)S layer prepared by the CSD method. This result could be evidence for the fact that ion-by-ion growth dominant mechanism during the CSD process. Despite the beneficial effect of using the (Cd,Zn)S buffer layer, irrespective of the type of the method, this approach can restrict the use of (Cd,Zn)S for the mass production line due to the reduced deposition rate. This technical note with an equipment-wise approach can be used as a technological guideline for the manufacturers to further develop of wet chemical buffer layer deposition system for the CIGS PV production line. Apart from the statistical and microscopic analysis provided in this technical report, in-depth optoelectronic characteristics must be addressed along with a cost-effectiveness analysis for further development. 

## Figures and Tables

**Table 1:** Comparison between the chemical bath deposition (CBD) and chemical surface deposition (CSD) processes.

Item	CBD Process	CSD Process
Chemical consumption *	~20 liter m^−2^ [11]	~6 liter m^−2^
Heating method	Indirect (via electrolyte)	Direct
Flow type	Batch process	Semi-continuous
Deposition side	Double	Single
Scalability	Good	Limited

* Consumption for a single panel (shall be reduced when the panel numbers increase in the batch).

**Table 2:** Properties of buffer layers formed on indium tin oxide (ITO)-coated glass by different deposition methods (CBD and CSD) using CdS and (Cd,Zn)S bath solutions.

Method	Buffer Layer	Process Time (min)	Properties		
			Thickness ^1^ (nm)	Transmittance ^2^ (%)	Zn/(Cd+Zn) ^3^
CBD	CdS	16	70.42 ± 2.04	78.62 ± 2.27	0
	(Cd,Zn)S	20	71.19 ± 3.47	84.79 ± 3.81	0.38 ± 0.04
CSD	CdS	12	71.18 ± 2.45	77.74 ± 1.12	0
	(Cd,Zn)S	16	72.37 ± 4.57	81.74 ± 2.96	0.27 ± 0.03

^1^ Thickness of the layer measured using an interferometer. ^2^ Average transmittance of the layer measured by ultraviolet-visible (UV/Vis) spectroscopy (wavelength: 300–1000 nm). ^3^ Calculation based on the compositional analysis by energy-dispersive X-ray spectroscopy (EDX) measurements.

**Table 3:** Compositional analysis of the surface of buffer layers on CIGS using EDX. Note that pieces from the CIGS substrate were obtained immediately after buffer layer deposition for the measurements (see Figure S2).

Method	Cd	S	Zn	Zn/(Cd + Zn)
CBD (Cd,Zn)S	42.69	45.79	11.53	0.20
CSD (Cd,Zn)S	47.96	49.91	2.13	0.04

**Table 4:** Effect of chemical solution type on CIGS PV panel performance; conversion efficiency (*Eff.*), fill factor (*FF*), cell open-circuit voltage (*V_OC/cell_*), current density (*J_SC_*), and series and shunt resistivity (*R_s_* and *R_sh_*).

Method	Buffer layer	*Efficiency*^1^ (%)	*FF* (%)	*V_OC/cell_* (V)	*J_SC_* (mA/cm^2^)	*R_s_* (Ω cm^2^)	*R_sh_* (Ω cm^2^)
CBD	CdS	11.71	61.97	0.625	30.33	9.10	1123.02
	(Cd,Zn)S	12.12	59.58	0.651	31.67	12.20	1882.13
CSD	CdS	11.88	63.55	0.633	30.98	8.77	1291.09
	(Cd,Zn)S	12.22	65.01	0.635	31.13	8.59	2185.53

^1^ Light conversion efficiency under AM1.5 equivalent illumination.

## Supplementary Materials

The following are available online at https://www.mdpi.com/1996-1944/13/7/1622/s1, Figure S1: AES depth profile of (Cd,Zn)S thin films grown on ITO-coated glass substrates, Figure S2: CIGS panel photo after the buffer layer deposition process and SEM image of the scraped CIGS piece, Figure S3: TEM images of AZO/i-ZnO/(Cd,Zn)S/CIGS interfaces with buffer layers prepared by different methods with energy-dispersive spectroscopy line-scans across the interfaces.
