# Supplementary material for: A Comparative Study of (Cd,Zn)S Buffer Layers for Cu(In,Ga)Se2 Solar Panels Fabricated by Chemical Bath and Surface Deposition Methods
Source: Materials (Basel). 2020 Apr 1;13(7):1622. doi: 10.3390/ma13071622 (PMC7178398; doi:10.3390/ma13071622)
Supplement: Supplementary file 1 [file materials-13-01622-s001.pdf]

materials

Supplementary Information for:

A Comparative study of (Cd,Zn)S buffer layers for Cu(In,Ga)Se_2 solar panels fabricated by chemical bath and surface deposition methods

Dowon Bae[1,2*]

[1] Delft University of Technology, Department of Chemical Engineering - Materials for Energy Conversion and Storage (MECS), Delft 2629HZ, The Netherlands; d.bae@tudelft.nl, bae.dowon@yahoo.co.kr

[2] LG Innotek R&D Center, 55, Hanyang Daehak-ro, Sanggrok-gu, Ansan, Gyeonggi, 426-791, Korea

* Corresponding author

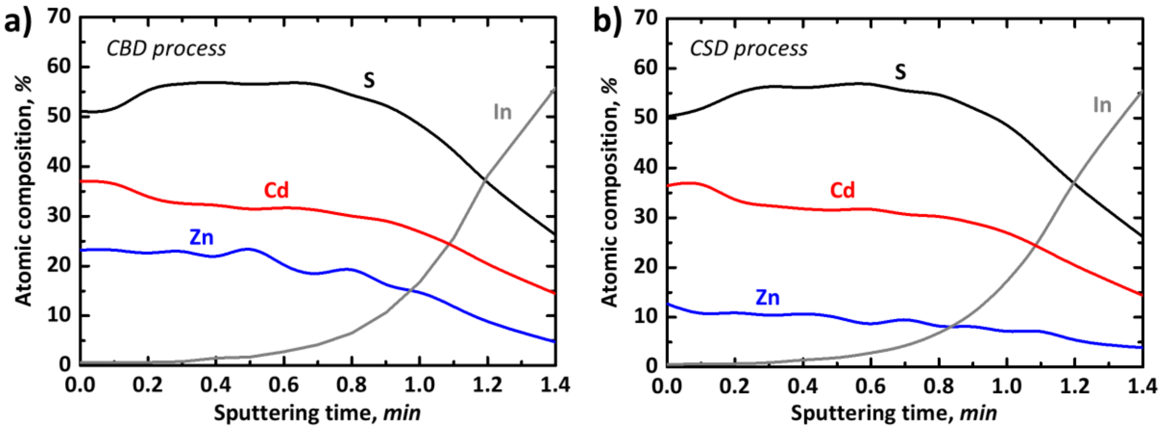

Figure S1 AES depth profile of (Cd,Zn)S thin films grown on ITO-coated glass substrates by the CBD (a) and CSD (b) processes.

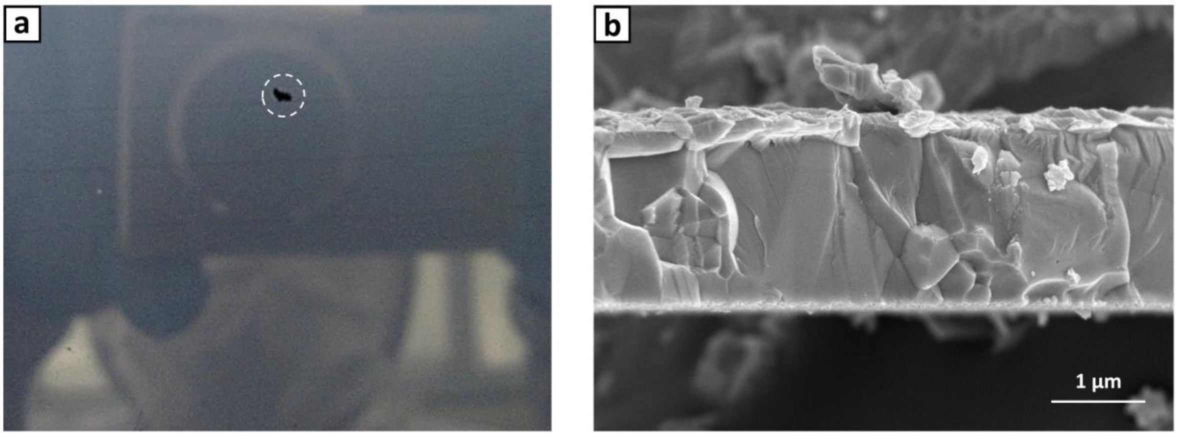

Figure S2 CIGS panel photo after the buffer layer deposition process (a). A small piece of the sample (SEM in b) was scraped from the panel by using a razor blade (dashed circle) for the EDX measurement.

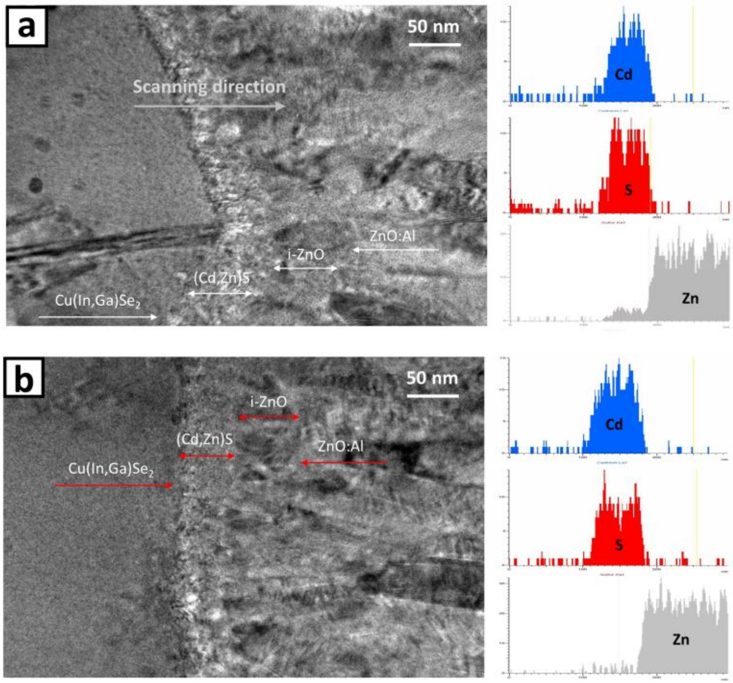

Figure S3 TEM images of AZO/i-ZnO/(Cd,Zn)S/CIGS interfaces with buffer layers prepared by different methods (CBD – a; CSD – b). The energy-dispersive spectroscopy line-scans across the interfaces are also displayed on the right side.

© 2020 by the authors. Submitted for possible open access publication under the terms and conditions of the Creative Commons Attribution (CC BY) license (http://creativecommons.org/licenses/by/4.0/).
